# Contemporary X-ray electron-density studies using synchrotron radiation

**DOI:** 10.1107/S2052252514018570

**Published:** 2014-08-29

**Authors:** Mads R. V. Jørgensen, Venkatesha R. Hathwar, Niels Bindzus, Nanna Wahlberg, Yu-Sheng Chen, Jacob Overgaard, Bo B. Iversen

**Affiliations:** a Center for Materials Crystallography, Department of Chemistry and iNANO, Aarhus University, Langelandsgade 140, Aarhus C, DK-8000, Denmark; b ChemMatCARS, Advanced Photon Source, University of Chicago, USA

**Keywords:** electron-density studies, synchrotron radiation, X-ray diffraction

## Abstract

Synchrotron radiation has many compelling advantages over conventional radiation sources in the measurement of accurate Bragg diffraction data. The variable photon energy and much higher flux may help to minimize critical systematic effects such as absorption, extinction and anomalous scattering. Based on a survey of selected published results from the last decade, the benefits of using synchrotron radiation in the determination of X-ray electron densities are discussed, and possible future directions of this field are examined.

## Abstract 2

The use of synchrotron radiation for experimental electron-density determination during the last decade is reviewed. Possible future directions of this field are examined.

## 1. Introduction   

Since the first X-ray diffraction experiments more than a century ago, the field of crystallography has advanced with tremendous speed. This evolution has been driven by better hardware, by exponential growth in computing power, and especially by better and brighter X-ray sources. Today, synchrotron radiation (SR) sources are available all over the world, and what started as a parasitic use of high-energy physics accelerators has become an essential probe for research in many scientific fields.

The electron density (ED) is arguably the most information-rich observable and, remarkably, it is available from rather simple X-ray diffraction experiments. The field of modern X-ray ED analysis commenced with the introduction of atom-centred multipole models in the 1970s (Stewart, 1976 ▶; Hirshfeld, 1977 ▶; Hansen & Coppens, 1978 ▶) and has, in line with other crystallographic fields, expanded its scope and capabilities as new technological advances have been made. SR has many compelling advantages compared with conventional X-ray sources, most of which can yield a significant benefit for accurate diffraction experiments. However, only a minor fraction of the published ED literature has been based on experiments using SR. In this short review, we will discuss the advantages and drawbacks of synchrotron radiation for ED studies based on results published during the last decade. A review of the field prior to 2004 was published by Coppens *et al.* (2005 ▶).

This paper is divided into six main sections. The first introduces the conventional ED formalism and discusses the data requirements. The following three sections describe recent results and developments in ED research using SR for organic compounds, metal–organic compounds and extended inorganic compounds, respectively. The fifth section presents recent work on refining multipolar models against high-resolution powder diffraction data, followed by a short general discussion of the field and an outlook.

## 2. Electron-density models and experimental considerations   

The spherical neutral atoms of the independent atom model (IAM) do not permit a detailed description of the electron distribution, so an analysis of the bonding beyond bond lengths is not meaningful. Instead, a model describing the redistribution of the valence electrons upon bonding is needed. The most widely used model for this is the Hansen–Coppens multipolar model (MM; Hansen & Coppens, 1978 ▶)
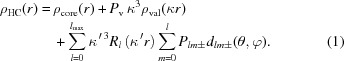
Here, the density is described as a sum of pseudo-atomic densities. Each of these is composed of a spherical core, a spherical valence shell and a set of spherical harmonic functions to describe the aspherical ED. The number of valence electrons (*P*_v_ + *P*_00_) and the contraction/expansion (κ, κ′) of the valence shells are allowed to vary in a least-squares refinement against the experimental data. The radial dependence of the valence shells (*R*_*l*_) is typically described by single-ζ Slater functions. This model deconvolutes the thermal motion and the density model, yielding the static ED. The thermal model relies on harmonic and/or anharmonic potentials. It is important to verify that an appropriate model is being applied and that the deconvolution of ED and thermal motion is satisfactory, *e.g. via* the Hirshfeld rigid-body test (Hirshfeld, 1976 ▶), residual density or the probability density function (Herbst-Irmer *et al.*, 2013 ▶). The resulting density is commonly analysed through the use of the deformation density (Δρ = ρ_MM_ − ρ_ref_), showing the density deformation with respect to a reference model, often the IAM.

Another widely used analysis framework is the quantum theory of atoms in molecules (QTAIM) developed by Bader (1990 ▶). In this theory, the interactions of the atoms are linked to the topological properties of the density. Most importantly, bond paths are defined by the gradient trajectories from neighbouring atoms and terminate at a saddle point, a so-called bond-critical point (BCP). The existence of a bond path is a necessary condition for a direct interaction between two atoms. However, it must be noted that this interaction may not necessarily be attractive, as shown *e.g.* between hydride ions in LiH (Gatti, 2005 ▶). This theory also provides a unique partitioning scheme, dividing the total density into open sub­systems referred to as atomic basins. A range of properties can be evaluated for the individual basins, *e.g.* net atomic charges, atomic volumes and electrostatic moments. Besides the density, another scalar field commonly used in the ED analysis is the Laplacian, 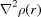. This function recovers the shell structure of the atoms and reveals local accumulation/depletion in the ED. For transition metals the outermost valence shell is not resolved, which complicates the analysis (Sagar *et al.*, 1988 ▶). The values of the Laplacian and of the ED at the BCP are commonly used to characterize the nature of chemical bonding (Bader & Essén, 1984 ▶; Espinosa *et al.*, 2002 ▶; Macchi *et al.*, 1998 ▶; Gatti, 2005 ▶). An attractive feature of the QTAIM is that it relies on the ED only, *i.e.* theoretical and experimental models can be compared directly.

The data requirements for ED modelling are quite simple but experimentally challenging, requiring high accuracy and precision up to a high resolution, often to (sinθ/λ)_max_ > 1.1 Å^−1^. Precision and accuracy are needed since the valence electrons only constitute a small fraction of the total electrons, and thus only a minor part of the intensity contains the chemically most important information. To attain high accuracy, it is important to minimize systematic effects present in the experiment. By minimizing these effects it is possible to apply a better correction and, in turn, to avoid systematic errors. To increase the precision, it is important to measure intensities with a high signal-to-noise ratio. High resolution is required for two reasons. The core electrons are tightly bound and scatter X-rays efficiently to high angles. At the same time, they are only slightly perturbed by bonding, and thus the use of high-resolution data allows for a better deconvolution of bonding features and thermal motion. To measure data to high resolution, it is often necessary to cool the crystal to liquid-nitrogen temperatures, or preferably even lower (Larsen, 1995 ▶; Iversen *et al.*, 1996 ▶, 1997 ▶). This also ensures that only the lower vibrational energy levels, which are more harmonic and thus better described by the commonly used thermal models, are occupied. A practical aspect is the added number of parameters in an MM ED model, commonly up to 34 parameters per atom, compared with a corresponding IAM with nine parameters per atom. Clearly, an MM requires a larger number of observations to maintain a suitable data-to-parameter ratio.

SR has many virtues that make it an ideal probe for ED experiments, namely a high and tuneable photon energy and an intensity which is many orders of magnitude higher than a sealed X-ray tube. The high photon energy minimizes the crystal absorption and contracts the projection of reciprocal space, leading to higher coverage with fewer detector settings. The tuneable energy is important to avoid absorption edges and thereby minimize anomalous scattering. The higher photon energy will also help to minimize extinction. This is discussed in more detail in §5. Furthermore, the nature of the source and the high quality of the monochromators and optics deliver a highly monochromatic beam, which simplifies peak integration.

The high intensity of the beam allows for smaller samples to be measured, which also helps to minimize systematic effects such as absorption and extinction. In addition, it also facilitates the measurement of weak high-order reflections not feasible using a conventional source. The high intensity also leads to faster experiments; just hours at a synchrotron *versus* days or even weeks at a conventional source. It is therefore feasible to collect complete and highly redundant data. Collecting highly redundant data, *n* > 5–10, enables better correction of systematic errors and allows for outlier rejections based on the distribution of the symmetry-equivalent reflections (Blessing, 1997 ▶; Jørgensen *et al.*, 2012 ▶). This is especially important for the correction of multiple scattering, which is a potential problem due to the use of shorter wavelengths at synchrotrons.

The use of SR also has some disadvantages that must be addressed. Often these are not discussed in the literature and the following list is based mainly on the authors’ personal experiences (Jørgensen, 2011 ▶; Schmøkel, 2013 ▶). The dis­advantages include beam instability, inhomogenous beam profile, crystal decay and inadequate detector technology. The beam stability used to be a problem on first- and second-generation storage rings, but contemporary third-generation facilities have a high reliability with more than 97% beam delivery. Furthermore, most facilities use a top-up filling pattern, thus avoiding exponential decay of the beam intensity. However, even with this high reliability, SR is still not as reliable as a conventional sealed X-ray tube. The availability of beam time has never been better, but many of the available single-crystal instruments are general-purpose instruments not necessarily optimized for high-resolution and high-accuracy ED experiments. One of the potential problems is the narrow spatial distribution of the often focused beam. This leads to intensity variations *versus* rotation angle as the crystal is not continuously fully immersed in a homogenous beam. These errors need to be corrected through an empirical correction routine, and although this significantly improves the internal consistency (Schulz *et al.*, 2009 ▶) it is a correction that could introduce systematic errors in the data. An additional complication is spatial movement of the beam. This can have a dramatic effect, especially if the amplitude is comparable with the size of the homogeneous part of the beam. Fast data acquisition implies that the reflecting condition is only fulfilled for a very short time. If the frequency of the spatial fluctuations is on the same timescale as the Bragg condition, this can lead to large intensity discrepancies between symmetry-equivalent reflections. It is hard to generalize how profound this effect is, as it depends greatly on the design of the beamline optics.

By defocusing the beam and shaping it using slits, it is possible to obtain a beam resembling a top hat, with a plateau of constant intensity. However, this approach significantly reduces the incident intensity and thus it is trade off that may be unacceptable for minute samples and/or weakly diffracting compounds. On the other hand, a slower experiment may cancel out the effect of spatial fluctuations of the beam by averaging, thus leading to better internal agreement. It is important to stress that obtaining the highest data quality is of utmost importance.

Another potential problem is that the detectors used at various beamlines are often identical or very similar to the detectors used at laboratory X-ray instruments, despite the much more intense short-wavelength source. Due to the shorter wavelength and thus smaller scattering angles, more reciprocal space can be covered with one detector setting. The result is that both intense low-order and weak high-order reflections are collected simultaneously. However, the dynamic range of the detector is often insufficient to collect meaningful data. This is not a problem for large unit-cell structures such as proteins, where the intensity is distributed among millions of Bragg reflections, but for a small unit-cell structure this poses a serious challenge. The intense inner reflections might saturate the detector, while the intensity of the weaker high-order reflections is barely above the background level. To circumvent this issue, it is often necessary to collect data using two (or more) different exposure times and subsequently scale the data to obtain a higher dynamic range of the combined data. This requires great care to ensure that there are enough data measured at a reasonable intensity over a wide range of resolutions in both data sets to facilitate their mutual scaling. Collecting the weaker parts of the data will mean deliberately overflowing the high-intensity reflections, but this can lead to difficulties in estimating the profiles of the reflections and great care needs to be taken during the integration. This is also true due to the fact that the profile obtained using SR is often more Lorentzian than the Gaussian shape often obtained at a conventional X-ray source. Overall, one has to realise that bonding effects are subtle and the precision of the intensity recording has to be at least a few percent, so good detector calibration is compulsory. This step can be combined with the data reduction, although it is better performed separately to avoid unnecessary correction steps. The requirements on the ED data in terms of both accuracy and precision are simply much more severe than in typical ‘structural’ studies.

## 3. Organic compounds   

Organic molecular compounds are a good realisation of the ideally imperfect crystal, and often extinction and absorption are small or even insignificant effects. Furthermore, the scattering contribution from the bonding electrons is relatively large compared with the contribution from the core electrons. Therefore, if crystals of high quality and a suitable size are available, it is possible to conduct highly accurate ED studies using a conventional X-ray source. This was shown in a comparison study between a Mo *K*α and an SR data set (Messerschmidt, Scheins & Luger, 2005 ▶). The two data sets were collected at similar temperatures (25 and 15 K, respectively) and show comparable signal-to-noise ratios, resolution and completeness. Although the two data sets are highly similar, the authors conclude that the conventional data are of higher quality. The redundancy of the conventional data is double that of the synchrotron data, which may influence the results in favour of the conventional data. However, the study establishes that conventional data are at least on a par with synchrotron data in fortunate cases of well behaved samples.

**Figure 1:**
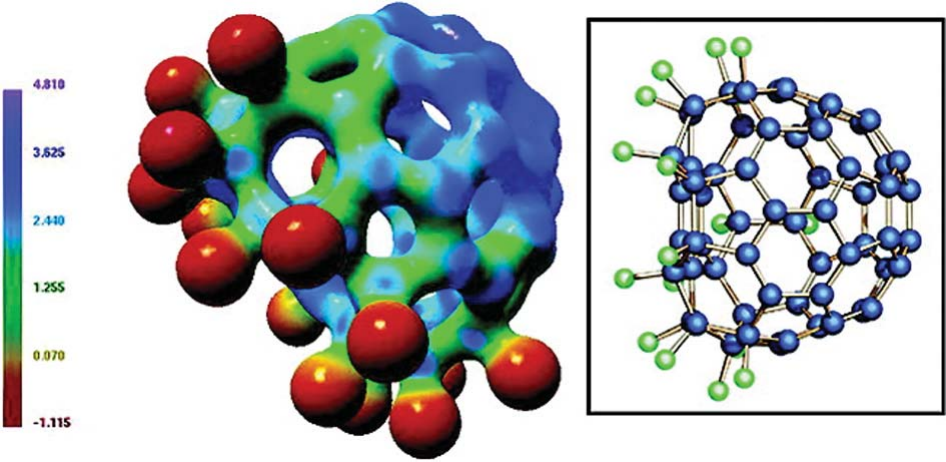
(Left) Experimental electrostatic potential (EP) of C_60_F_18_ mapped onto the ED isosurface at ρ = 0.8 e Å^−3^. Negative EP distributions (dark red) appear in the region of the fluorine substituents, while the positive regions (dark blue) cover the opposite non-halogenated C_60_ surface. (Right) Molecular structure of C_60_F_18_. Reprinted with permission from Luger *et al.* (2005 ▶). Copyright (2005) American Chemical Society.

Even though data of a quality comparable with SR data can be routinely collected at a conventional source, there are some advantages of SR for organic molecular crystals, namely that the intensity is orders of magnitude higher than that from a laboratory source. This enables the experimenter to use smaller samples, collect data to a higher resolution (at a higher significance) or collect more redundant data in a shorter time. An example of the high resolution possible from organic crystals using SR was recently demonstrated by Nassour *et al.* (2014 ▶), where data up to (sinθ/λ)_max_ = 1.51 Å^−1^ were collected on 2-methyl-1,3-cyclopentanedione. A significant reduction in data-collection time was shown by Luger *et al.* (2005 ▶), where four high-resolution data sets on C_60_F_18_, C_60_Cl_30_, the base pair 9-methyladenine-1-methylthymine, and the tripeptide l-alanyl–glycyl–l-alanine were collected within a 12 h period (Fig. 1 ▶). The results obtained were compared with ‘regular’ high-redundancy ED data sets, and the ED and Laplacian at the BCPs were in accordance with the fast screening data sets (Hübschle, Scheins *et al.*, 2007 ▶; Checińska *et al.*, 2006 ▶). Two of the crystals above are fullerene derivatives, which often display poor quality and disorder due to the high mobility of the nearly spherical molecules. Experiments using SR have been essential in determining the EDs of a range of these systems (Wagner *et al.*, 2002 ▶; Kalinowski *et al.*, 2010 ▶; Grabowsky *et al.*, 2010 ▶).

ED investigations are widely used to understand chemical bonding in terms of topological characterizations. In [1.1.1]propellane the presence of an unusual C⋯C interaction was observed between the bridgehead carbon atoms (Messerschmidt, Scheins, Grubert *et al.*, 2005 ▶). Even though this C⋯C interaction has the characteristic distance of a typical covalent bond, there was no charge accumulation in the bond-critical point, ρ_BCP_ = 1.31 (3) e Å^−3^, as revealed by a positive value of the Laplacian, 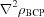 = 10.3 (1) e Å^−5^. The observed experimental results were supported by theoretical calculations in quantitative agreement. The authors noted that the study was only feasible with SR since only weakly diffracting crystals could be obtained.

Another successful application of EDs is in the study of chemical reactivity in solids. In order to understand charge-transfer phenomena *via* dative N—B bonds and dihydrogen (H⋯H) contacts in Lewis acid–base adducts, ED models from both experiment and theoretical calculations were obtained (Mebs *et al.*, 2010 ▶). The dative N—B bond was found to have both covalent and ionic bond properties, with a charge transfer of 0.03–0.14 electrons in the molecular adducts. It was further demonstrated that short H⋯H contacts play a crucial role in the charge-transfer process between Lewis acid–base adducts.

The evaluation of intermolecular interactions and electrostatic properties has been one of the focal points of ED studies of organic systems. Conventional hydrogen bonds (*e.g.* O—H⋯O, O—H⋯N, N—H⋯O and C—H⋯O) and weak intermolecular interactions (*e.g.* C—H⋯π, π⋯π and H⋯H) in molecular crystals have been explored by ED determination using SR (Meents *et al.*, 2008 ▶; Scheins *et al.*, 2007 ▶; Mebs *et al.*, 2010 ▶; Checińska *et al.*, 2011 ▶; Małecka *et al.*, 2013 ▶; Zhurov & Pinkerton, 2013 ▶). The derived topological properties and interaction energies were used to classify them as strong, weak or very weak intermolecular interactions. As mentioned above, a very high-resolution data set was collected by Nassour *et al.* (2014 ▶) on 2-methyl-1,3-cyclo­pentanedione. This compound forms strong intermolecular O—H⋯O hydrogen bonds, which are presumably of the resonance-assisted hydrogen bond type (RAHB; Gilli *et al.*, 1989 ▶). The topological analysis confirms that the hydrogen bond is consistent with the RAHB model proposed by Madsen *et al.* (1998 ▶), having high and equal negative charges on the donor and acceptor (−1.26 and −1.09, respectively) and a substantial positive charge on the hydrogen (0.35 e). The effect is much less pronounced than seen for the intra­molecular RAHB in benzoylacetone, where the Laplacian at the O—H BCP is negative, indicating the covalent character of the bond (Madsen *et al.*, 1998 ▶). A word of caution may be appropriate with respect to hydrogen-bonding studies. It has been established that, particularly in the case of studies of strong hydrogen bonds, the use of combined X-ray and neutron data (X–N procedure; Coppens, 1967 ▶; Blessing, 1995 ▶) is important for obtaining accurate EDs (Overgaard *et al.*, 1999 ▶). Currently, the number of X–N ED studies is very low, but this may change with the very powerful new single-crystal neutron diffractometers coming online at the latest spallation sources (Jørgensen *et al.*, 2014 ▶).

Studies of weak interactions in biologically important compounds have also contributed to understanding the mutual recognition interactions at the active sites of biological systems, which are key issues in life science (Wagner *et al.*, 2004 ▶; Luger, 2007 ▶; Dittrich *et al.*, 2007 ▶). Here it is important to highlight the work done validating the transferability of multipole parameters (Dittrich *et al.*, 2000 ▶, 2003 ▶; Checińska *et al.*, 2006 ▶; Scheins *et al.*, 2007 ▶; Johnas *et al.*, 2009 ▶), which in part was accelerated by the high throughput enabled by SR and which has improved both the determination of the electrostatic properties of enzyme–ligand binding sites of macromolecules and macromolecular crystallography in general (Dittrich *et al.*, 2007 ▶; Guillot *et al.*, 2008 ▶; Lecomte *et al.*, 2008 ▶). A recent example of an experimental ED study of a protein–ligand complex is that of human aldose reductase and an inhibitor, fidarestat, of said protein (Fournier *et al.*, 2009 ▶). Here, the ED of the fidarestat molecule was modelled by MM refinement and the ED of the protein was constructed from the ELMAM database (Zarychta *et al.*, 2007 ▶). From the electrostatic potential (EP), a clear ‘key–lock’ interaction was identified for the inhibitor and the binding site on the protein.

Evaluation of the EP was also employed by Clausen *et al.* (2011 ▶, 2014 ▶) in the study of guest–host effects in β-hydroquinone frameworks, by the introduction of polar molecules into the cavities of the structure. For the empty framework a minute sample (∼20 µm) was used, as larger samples showed merohedral twinning, and thus the scattering power of the small sample could not be measured at a conventional X-ray source. To study the perturbation of the ED upon the inclusion of guest molecules, a crystal of β-hydroquinone with acetonitrile was prepared. This system displays pseudo-symmetry, with a nearly centrosymmetric host but non-centrosymmetry of the guest molecules. This leads to a group of very weak reflections, which could not be collected using conventional X-ray sources. Even with bright SR available from an undulator at a third-generation synchrotron source, it was not possible to collect these reflections with sufficient quality and significance to allow unrestrained ED modelling. However, an important result of that work, confirmed by theoretical calculations, was that the inclusion of a polar guest significantly polarizes the host. This effect is not accounted for in *e.g.* molecular mechanics simulations, where nonpolarizable force fields are normally applied to derive the interaction energies.

**Figure 2:**
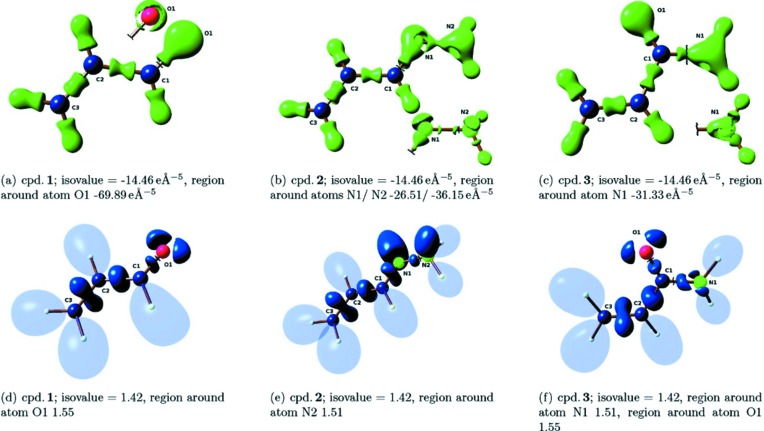
Isosurface representations of the Laplacian of (*a*)–(*c*) the ED and (*d*)–(*f*) the ELI for three model compounds studied by Grabowsky *et al.* (2011 ▶). Compound **1**: propenal, **2**: (*E*)-allylidenehydrazine and **3**: acrylamide. The ELI conveys more immediate information about reactivity than the Laplacian of the ED. Reprinted with permission from Grabowsky *et al.* (2011 ▶). Copyright (2011) American Chemical Society.

The EP is also very useful in differentiating the activity of molecules, where topological analysis of the ED and the Laplacian does not provide unambiguous answers. This approach was successful in elucidating the activity of two pencillin derivatives, namely the active penamecillin and the inactive penamecillin-1β-sulfoxide (Wagner *et al.*, 2004 ▶). The EP maps show that the entire phenylalanyl residue is negatively polarized in penamecillin-1β-sulfoxide, whereas this is not the case in the active penamecillin. Additionally, the negative potential extends over one side of the lactam ring and blocks the site of nucleophilic attack on the carbonyl bond in the inactive form. Another successful ED study of chemical reactivity by Grabowsky *et al.* (2011 ▶) was performed on a series of α,β-unsaturated carbonyl and hydrazine molecules. Reactivity differences of the carbon atom at the β-position arise due to the opposing resonance effects of the electron-withdrawing carbonyl group and the electron-donating hydrazone group. By analysis of the EP, the Laplacian and the electron localizability indicator (ELI) (Fig. 2 ▶), it was demonstrated that a nucleophile could attack the carbon atom at the β-position in the α,β-unsaturated carbonyl compound, whereas the same position would be reactive towards an electrophilic attack in the α,β-unsaturated hydrazone compound. The ELI is not commonly available experimentally, but X-ray constrained wavefunction fitting (XCW; Jayatilaka, 1998 ▶; Jayatilaka & Grimwood, 2001 ▶) enabled the authors to estimate this descriptor experimentally.

## 4. Metal–organic compounds   

SR is relatively rarely used in ED studies of metal–organic compounds. The explanation may well be that crystals of sufficient scattering power for use with conventional sources are available and that extinction and/or absorption effects are not particularly severe. Nevertheless, significant advantages accompany the exploitation of the increased intensity even in such cases, as will be highlighted by the following examples. For the majority of the studies presented here, a key factor is the simultaneous use of liquid He cooling to reach crystal temperatures below 30 K. This serves a dual beneficial purpose by reducing the effects of thermal motion and minimizing thermal diffuse scattering (TDS). The latter is a systematic effect that originates from one-phonon scattering and coincides with the Bragg position, leading to, if un­corrected, overestimation of intensities. It is very rarely corrected for, as it requires accurate lattice dynamic calculations, but it primarily affects the thermal parameters (Willis & Pryor, 1975 ▶).

One category of compounds where SR has played an essential role in ED studies are the metal–organic framework (MOF) extended structures, also commonly referred to as coordination polymers. In one study two isostructural compounds, *M*_3_(C_8_H_4_O_4_)_4_(C_5_H_11_NO)_3_(C_4_H_12_N)_2_, *M* = Co, Zn, were only obtained as tiny crystallites of the order of 10–30 µm, and their small size prevented even a simple structure determination using a conventional source (Clausen *et al.*, 2008 ▶). Magnetic susceptibility measurements of the Co compound (three Co cations are connected by bridging carboxylate groups) suggested a ferromagnetic ordering at temperatures between 42 and 85 K and an antiferromagnetic ordering at even lower temperatures. The study provided a model of the ED, and a QTAIM analysis did not recover BCPs between any of the metal atoms. Instead, two Co—O bonds, including that to the bridging μ_2_-coordinating oxygen, differed from the others by possessing significant covalent character. On the basis of these observations, these atoms and bonds were singled out as potential paths for the magnetic inter­action.

The ED in two Mn-containing carboxylate-bridged co­ordination polymers with different dimensionalities has been studied using SR by the same group. Two structurally very similar compounds with different solvent molecules, di­methyl­formamide (DMF; Poulsen *et al.*, 2004 ▶) and diethylformamide (DEF; Poulsen *et al.*, 2005 ▶), they both have Mn chains along the *a* axis with direct Mn⋯Mn separations of around 3.5 Å and significantly longer inter-chain distances. The channels created by the chains contain the solvent molecules. The magnetic properties of the two systems are different: for DMF there is no ordering in the temperature range from 2–400 K, while the DEF analogue shows an antiferromagnetic ordering at 4 K. This was explained by differences in the chemical bonding in the two systems. The electron distributions of the metal ions, and derived properties such as the *d*-orbital populations (Holladay *et al.*, 1983 ▶) and atomic charges, were significantly different in the two compounds. In the DEF system the metal–ligand interactions contain covalent components, whereas the DMF system is highly ionic. This difference explains the antiferromagnetic ordering in the DEF system and the absence of ordering in the DMF system. The different chemical bonding also significantly alters the EP inside the cavities of the two systems, which exhibit opposite ‘polarities’. This is expected to make the molecular inclusion properties of these two MOFs significantly different. Previously, SR has also been used to study the guest-inclusion properties of large Cr-wheel complexes by Overgaard *et al.* (2002 ▶), and in that study the prediction of inclusion properties based on the experimental EP was firmly established by the synthesis of relevant host–guest complexes.

Two ED studies concern an isostructural series of simple formate-based MOFs exhibiting rich magnetic properties, *M*(HCOO)_2_(H_2_O)_2_ (Poulsen *et al.*, 2007 ▶; Jørgensen *et al.*, 2013 ▶). The study of the *M* = Mn system featured a comparison of three different data sets and it was concluded that the powerful combination of SR data and very low temperatures provides the most accurate data (Poulsen *et al.*, 2007 ▶). These MOFs contain two kinds of octahedral *M* cations, one bonded only to formate groups, and the other to two formates along the *c* axis and four water molecules in the *ab* plane. This produces a pseudo two-dimensional structure where every second layer in the *ab* plane is made up of formate-bridged *M* cations interspersed with layers of non-connected *M* cations. From the atomic charges and *d*-orbital populations in the Mn system, it was concluded that the Mn cations are high-spin entities with five unpaired electrons, in agreement with measurements of the low-temperature magnetic susceptibility. Comparison of the Mn system with the ED of the non-magnetic *M* = Zn reference compound identified potential magnetic interaction pathways through the hydrogen bonds and the carboxylate groups (Jørgensen, 2011 ▶; Jørgensen *et al.*, 2013 ▶). In the *M* = Zn study, both synchrotron and conventional X-ray diffraction were measured. The synchrotron data led to an ED model similar to the conventional data, although for some unknown reason the atomic displacement parameters were much smaller than expected. The conventional data were therefore used in the analysis.

**Figure 3:**
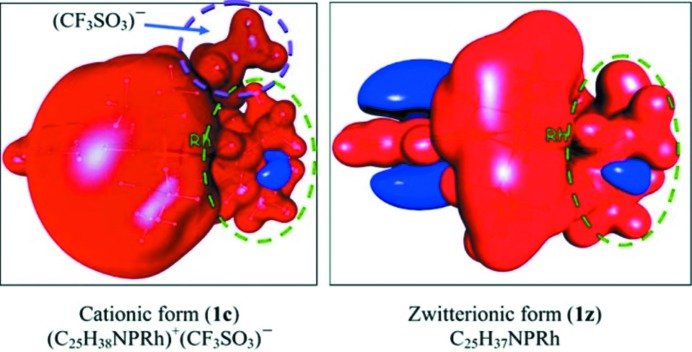
Experimentally determined molecular EP at an isosurface with |*V*(*r*)| = 0.05 a.u., showing the similarity of the form of the EP around the Rh atom (red denotes negative EP and blue positive EP). Reprinted with permission from Bendeif *et al.* (2012 ▶). Copyright (2012) American Chemical Society.

A challenging 30 K ED study using SR on two related Rh-containing complexes was recently carried out by Bendeif *et al.* (2012 ▶). The challenge comes from the fact that rhodium is a second-row transition metal, with a large core scattering contribution. Below, we discuss in more detail the problems – along with possible solutions – associated with the ED of heavy-element systems. The aim of this particular study was twofold: firstly, to examine whether the ED could support the bonding formalisms commonly used, and secondly, to test if zwitterionic and cationic compounds have similar charge distributions near the Rh atom, thereby opening up the use of the former type of system in organometallic catalysis, where equivalent cationic Pt-containing compounds are efficient in catalysing substrate transformations. Both goals were achieved through a topological analysis of the density, while the illustrative power of the electrostatic potential on the molecular surface was used to show the similarity of the Rh atoms in the differently charged molecules (Fig. 3 ▶), thereby confirming, ‘for the first time, that appropriately designed zwitterionic complexes can effectively emulate the charge distribution found within ubiquitous cationic platinum-group metal catalyst complexes’.

Another use of SR to determine ED has been carried out by Grabowsky *et al.* (2009 ▶) on a molecular siloxane compound. Their object was to study the reactivity of the siloxane linkage, which is constituted by an Si—O—Si group, by means of combined topological analysis of the ED and electron localization function (ELF). The study represents one of the first implementations of the XCW fitting method, in order to derive an ELF that to some extent includes input from the experimental structure factors. The background of the study was that this particular Si compound exhibits an intermolecular hydrogen-bond type that is very rarely seen. Using the tools of both ELF and ED on *ab initio* models, the authors were able to quantify that, on decreasing the bond angle of the Si—O—Si part from 180° to 110°, the covalency of the Si—O bond, gauged by ED and the Laplacian at the BCP as well as by the delocalization index, increases, as does the basicity, and thereby the hydrogen-bond acceptor capabilities are enhanced. The observation that the Si—O—Si angle in the studied compound is significantly strained is in full accordance with these observations.

An SR study was carried out on a chemically interesting compound containing a direct chemical bond between two formally divalent Zn cations, the coordination spheres of which are completed by Cp* groups (Van der Maelen *et al.*, 2007 ▶). The molecule represents a growing body of complexes in which two metal atoms (also including the main group metals) are connected through a chemical bond. Of particular importance are the similar complexes which feature unusually low metal oxidation states. The latter have been intensely studied in recent years, not least due to their useful reactivity in solution. In this study, Van der Maelen and co-workers combined X-ray and neutron diffraction data, firstly to exclude the presence of hydridic H atoms bonded to the Zn cations, and secondly to obtain accurate C—H bond distances which were used as constraints for the multipolar refinement. However, since the temperatures of the two experiments differed by 70 K, knowledge of the H-atom atomic displacement parameters was not exploited to carry out an X–N refinement. The study provided clear evidence for a direct open-shell intermediate Zn—Zn chemical bond, while the atomic charge on the Zn was found to be 0.72 e.

Another example within this category was reported by Overgaard *et al.* (2008 ▶), who studied a compound having two Co atoms in close proximity bridged by a substituted formal ethyne group and coordinated to carbonyl groups. The pertinent question to answer here is whether a chemical bond exists between the two Co atoms or not. By analysis of the source function (SF; Bader & Gatti, 1998 ▶) and by the fact that no Co–Co BCP was found, it was unambiguously shown not to be the case. The formal 18-electron count suggests that in fact a bond should exist, but accompanying theoretical studies established that the ground-state structure is in fact characterized as a singlet bi-radical, which is fully in accordance with the lack of a chemical bond. In addition to this result, the study included an analysis of the achievable precision of the diffraction data. The molecule contains a CoC_2_ triangle, which experimentally (in contrast with theory) provides rather curved bond paths, nearly leading to a catastrophic situation as the ring critical point and one BCP are close to merging. By artificially adding noise to the theoretical structure factors, the near-catastrophic situation reappeared, illustrating that recovery of the true ED in such an environment is hampered by experimental noise, not by the restricted flexibility of the multipole model.

Mixed-valence polynuclear transition metal complexes constitute an important branch of chemistry and SR ED studies have provided important insight into the chemical bonding of such systems. The mixed-valence trinuclear carboxylates, [*M*_3_O(O_2_C*R*)_6_ *L*_3_]·*nS* (*M* = metal atom, *R* = organic group, *L* = terminal monodentate ligand and *S* = solvent molecule of crystallization) are examples of flat potential-energy surface systems where very subtle changes in the surroundings of the molecules (*e.g.* disappearing disorder in solvent molecules) can drastically change the molecular structure and properties. The systems exhibit intramolecular electron transfer and, by comparing the experimental EDs of different systems in an ED correlation approach, it has been possible to identify the electrons involved in the transfer process occurring in the Fe-containing analogue (Overgaard *et al.*, 2003 ▶, 2009 ▶). A peculiar feature of these systems is that, for the mixed-valence system, the hybridization of the central O atom appears to be *sp* ^3^-like, even though the central Fe_3_O group is planar. Another type of flat potential-energy surface system are the linear metal chain systems (Rohmer & Bénard, 2001 ▶), which among many other things have been studied as potential molecular wires (Joachim *et al.*, 2000 ▶). Some of the materials exhibit temperature-dependent spin cross-over and a highly intriguing structural isomerism, with the presence of both symmetric and asymmetric molecules. A study was carried out of Co_3_(dpa)_4_Cl_2_ using both conventional and SR data. The crystals were prepared shortly before the conventional experiment and had unfortunately lost some solvent molecules before the synchrotron experiment. Therefore only the conventional data had sufficient quality to establish a reliable ED (Poulsen *et al.*, 2009 ▶).

One of the explanations for the lack of ED studies based on SR data for this class of compound may originate in the observation that metal–organic compounds (in contrast with purely organic or hard inorganic compounds) are often prone to crystal decay when inserted in the extremely intense synchrotron X-ray beam. This happens despite the low temperatures generally used in the experiments and major efforts have been devoted to solving this problem, but even the use of thick metal foils to attenuate the direct beam, off-setting the mirrors slightly to reduce peak intensity and other efforts have often been in vain. To the best of our knowledge, this problem persists. It could be imagined that the problem might be minimized by the use of more sensitive detectors that enable a faster execution of the experiment, as speculated in the *Outlook* (§7).

## 5. Extended inorganic materials   

Arguably, the most difficult class of materials for experimental ED determination are the extended inorganic compounds, including minerals (Schmøkel *et al.*, 2013 ▶). The challenges are intrinsically tied to the samples themselves. Weak intermolecular forces hold molecular crystals together, whereas inorganic solids are often formed by strong interactions. Therefore inorganic solids are often highly perfect with a low mosaicity and, combined with the large scattering power which in part comes from the high density of the materials, they may be severely affected by extinction effects. Additionally, many inorganic compounds contain heavy elements, which significantly increase the absorption and render it more difficult to probe the valence electrons, as they only constitute a minute fraction of the total electron count. The often small and highly symmetric unit cells also pose a problem, as the smallest sampling frequency of a simple cubic unit cell is 1/|*a*|. Delocalized electrons or diffuse valence electrons from heavier elements are contracted in reciprocal space and thus information about these is only available in a limited number of low-angle reflections. However, in contrast with these significant experimental difficulties, their high symmetry and often low number of atoms make inorganic materials attractive for theoretical investigations. It is often possible to use fully periodic boundary conditions and large basis sets to increase the accuracy.

**Figure 4:**
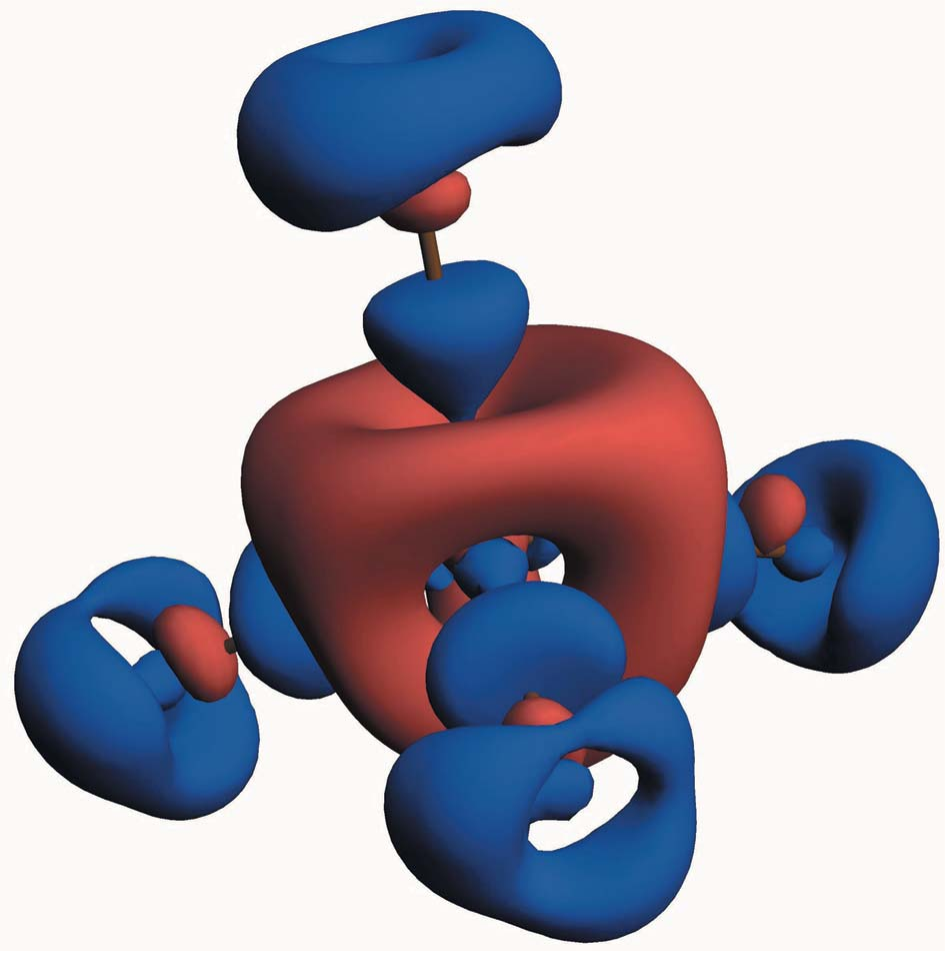
Three-dimensional static deformation density of the sulfate group in K_2_SO_4_ obtained from multipole refinement. Reprinted with permission from Schmøkel *et al.* (2012 ▶). Copyright (2012) American Chemical Society.

Crystallographic textbooks (Woolfson, 1997 ▶; Giacovazzo *et al.*, 2011 ▶) often note that repeated flash cooling can increase the mosaicity of a sample and thereby decrease the effect of extinction, but this treatment will also reduce the crystal quality and so should be avoided for accurate ED experiments. A better approach is to use a minute sample and a high-energy X-ray source. The best way to facilitate this is to use SR. A clear example of this was seen in the ED study of K_2_SO_4_ by Schmøkel *et al.* (2012 ▶), where ED experiments using conventional sources proved unfeasible due to severe extinction. An approximately 30 µm crystal was studied using a 30 keV synchrotron X-ray beam. In this data set the (020) reflection was the one most affected by extinction, but only 3.8% as refined against the MM. A testimony to the accuracy of the data is that it was even possible to refine the population of the very diffuse 4*s* electrons on potassium. The resulting density was compared with theoretical calculations and good correspondence was found. Analysis of the experimental and theoretical models dismissed the chemistry textbook concept of hypervalence in sulfate ions (Fig. 4 ▶). Using elaborate analysis of the SF, a bonding situation was observed where the S—O interactions can be characterized as highly polarized covalent bonds, with the ‘single-bond’ description prevailing significantly over the ‘double-bond’ picture.

**Figure 5:**
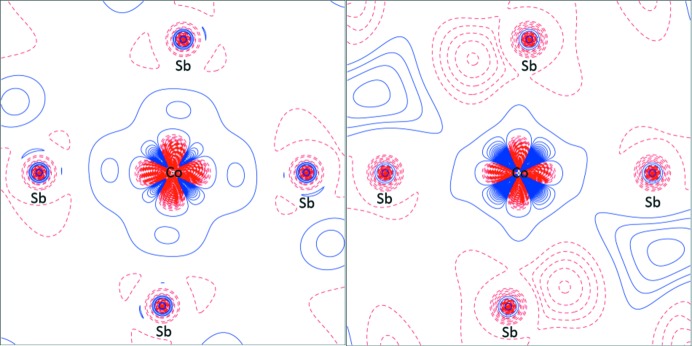
Contour plots of the static deformation density in the plane of the CoSb_4_ unit for (left) a multipole fit to theoretical data, and (right) a multipole fit to experimental data with κ parameters fixed at theoretical values.

An alternative approach to avoid extinction is to use quantitative convergent beam electron diffraction (QCBED), which gives extinction- and absorption-free structure factors on an absolute scale (Zuo, 2004 ▶). This technique is limited to low-order reflections and thus needs to be combined with X-ray data for a full ED refinement. The complementarity of the two techniques was shown by Streltsov *et al.* (2003 ▶) for α-Al_2_O_3_, where QCBED data were combined with synchrotron data from Maslen *et al.* (1993 ▶). The X-ray data showed significant extinction and improved residuals were found after including the electron diffraction data. QCBED is limited by the unit-cell volume and *e.g.* for CoSb_3_ with a cubic unit cell, *a* ≃ 9 Å, it was only possible to measure two reflections (Sæterli *et al.*, 2011 ▶). Since only two reflections were available, it was not possible to scale these to the X-ray data collected by Schmøkel and co-workers (Schmøkel, Bjerg, Overgaard *et al.*, 2013 ▶; Schmøkel, Bjerg, Larsen *et al.*, 2013 ▶). In this work, three synchrotron data sets were compared with a conventional data set. It was immediately seen that the conventional data were inferior to the three synchrotron data sets. This was due to the size of the sample and the lower photon energy, yielding a much higher absorption with a transmission of only 0.22 for the conventional data and around 0.95 for the synchrotron data. Additionally, extinction was quite severe for the conventional data, 49% for the (130) reflection, while the synchrotron data showed less than 0.5% reduction in the intensity of the same reflection. ED refinements against the best synchrotron data led to a rather high residual peak at the high-symmetry position (0, 0, 0), which accentuates another problem mostly encountered for inorganic materials, namely that noise tends to accumulate on high-symmetry positions (Cruickshank & Rollett, 1953 ▶; Rees, 1976 ▶). Comparison with the ED obtained from periodic density functional theory (DFT) calculations showed significant differences. By fixing the κ parameters to those obtained from MM refinement against the theoretical data, as suggested by Abramov *et al.* (1999 ▶) and Volkov *et al.* (2000 ▶), much better correspondence was found (Fig. 5 ▶). This shows that, even with state-of-the-art experiments, it may be necessary to cross-validate the results with other methods, in particular theoretical calculations, and free unconstrained refinement is not always possible.

Cross validation of theory and experimental EDs was also done by Gibbs and co-workers (Gibbs *et al.*, 2005 ▶, 2008 ▶; Kirfel *et al.*, 2005 ▶), where a range of *X*—O (*X* = Mn, Fe, Co, Mg, Na, Al, Be, Ca, Si, P, B) bonds in a series of minerals were investigated. Comparing the value of the density and the value of the Laplacian at the BCP, the authors assessed the agreement between experiment and theory. Good agreement was found for the data sets measured with SR. For a number of data sets collected with conventional X-ray sources, the agreement was worse than that found for the synchrotron studies, underlining the advantage of SR for these kinds of materials.

Recently, Oganov *et al.* (2009 ▶) presented a novel structure of a new polymorph of boron, γ-B_28_. The structure is composed of B_12_ icosahedral clusters and B_2_ dumb-bells arranged in an NaCl-type lattice. Based on theoretical calculations and subsequent integration of the atomic basins, Oganov *et al.* concluded that there is charge transfer from the dumb-bell to the B_12_ cluster, *i.e.* (B_2_)^δ+^(B_12_)^δ−^ with *δ* ≃ 0.5–0.6 e^−^, and the authors coined the term ‘boron boride’. The interpretation of the bonding with charge transfer between boron atoms caused some controversy, which was finally settled (Oganov *et al.*, 2011 ▶) in part by an experimental ED study revealing polar covalent bonds and charge transfer similar to that predicted by theory (Mondal *et al.*, 2011 ▶). Later, an experimental ED study of the α-B_12_ polymorph was undertaken by Mondal *et al.* (2013 ▶). Unlike the γ-polymorph, no charge transfer is observed in the α-form, but it was concluded that the orbital order of the B_12_ icosahedra is highly similar in both polymorphs and likely similar in other boron polymorphs and boron-rich solids (Mebs *et al.*, 2011 ▶; Mondal *et al.*, 2013 ▶).

Finally, another study of polymorphic structures, iron pyrite and marcasite, was recently published by Schmøkel *et al.* (2014 ▶). To minimize extinction, the samples used in the experiments were only approximately 10 µm. Due to the high-intensity X-ray beam it was possible to collect data to a resolution above 1.4 Å^−1^ for both crystals. Besides the experimental ED models, the corresponding theoretical models were also presented and compared. In general, excellent correspondence between the pairs of models was observed, except for the 4*s* population which was significantly lower in the experimental model of pyrite. This was attributed to the high symmetry of pyrite (space group 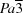), causing only two unique reflections to be below 0.2 Å^−1^. For marcasite (space group *Pnnm*), four reflections are present below 0.2 Å^−1^ and here a much better agreement between theory and experiment was found. For both structures, the bonding is neither ionic nor purely covalent but can be categorized as polar covalent interactions. This is supported by significantly lower atomic charges than the formal +2 and −1 charges expected in FeS_2_. The main difference between the two polymorphs is a more covalent S—S interaction and a correspondingly weaker Fe—S interaction in pyrite compared with marcasite. These observations are corroborated both by the local properties obtained from MM and QTAIM, and by a non-local perspective from band structure calculations (Schmøkel *et al.*, 2014 ▶).

In contrast with the materials discussed above, *i.e.* small unit cells, heavy atoms and highly perfect crystals, zeolites present very different challenges. Due to their large pores these crystals have limited scattering power, as well as large thermal motion and disorder between Al and Si. In a recent study by Porcher *et al.* (2014 ▶), the ED of an Na–*X* zeolite was presented. Both SR and conventional data were reported, but due to higher redundancy the conventional data were found to be superior to the SR data.

## 6. Electron density based on SR powder diffraction   

As described above, the diffracted intensities from highly crystalline inorganic extended materials may be severely affected by extinction. In these cases, synchrotron powder X-ray diffraction (SPXRD) has become a competitive alternative for measuring accurate structure factor amplitudes, |*F*_obs_| (Nishibori *et al.*, 2007 ▶; Svendsen *et al.*, 2010 ▶; Fischer *et al.*, 2011 ▶; Bindzus *et al.*, 2014 ▶). It also remains the only viable option if sufficiently large single crystals cannot be grown, although size restrictions are practically eliminated with present-day synchrotron flux. ED determination based on SPXRD data is inherently more challenging than the corresponding single-crystal case, as the three-dimensional information in the diffraction pattern has been projected into one dimension. The key problem in the data treatment is therefore to extract the structure factors from the pattern without introducing significant bias towards the extraction model. SPXRD yields data with negligible extinction and markedly reduced absorption, thus in some cases counterbalancing the added complications in the data analysis. Furthermore, data collection is very fast compared with single-crystal experiments and all data can be collected in a single exposure. The latter prevents systematic errors from merging a multitude of detector frames, each possessing a slightly different scale factor (Jørgensen *et al.*, 2012 ▶).

EDs from SPXRD are traditionally determined using the maximum entropy method (MEM). This statistical approach does not suffer from parameter correlation and is a powerful tool to probe complex structural effects, typically occurring in materials of technological interest (Takata, 2008 ▶; Nishimura *et al.*, 2008 ▶; Kim *et al.*, 2010 ▶; Maki *et al.*, 2013 ▶). MEM yields dynamic EDs, and this has made it challenging to interpret the results and extract quantitative measures of relevance for physics and chemistry. The QTAIM approach used in analysis of numerous static EDs obtained from mulipole modelling is not strictly valid for dynamic EDs (Iversen *et al.*, 1995 ▶). However, use of dynamic deformation densities still makes it possible to obtain important information (Bentien *et al.*, 2000 ▶), and semi-quantitative bonding descriptions can now be obtained routinely owing to recent methodological advances (van Smaalen & Netzel, 2009 ▶; Mondal *et al.*, 2012 ▶; Bindzus & Iversen, 2012 ▶; Prathapa *et al.*, 2013 ▶). On the qualitative level, valuable information about atomic connectivity, hybridization and orbital populations may be accessed (Kato *et al.*, 2003 ▶; Ohno *et al.*, 2007 ▶; Aoyagi *et al.*, 2008 ▶).

In recent years, the field of SPXRD has progressed to a stage where it is realistic to go beyond MEM estimations and perform highly accurate ED studies employing the atom-centred MM. Due to the inherent challenges of overlapping reflections and background subtraction, the method is still limited to high-symmetry compounds with small unit cells. In these cases, well resolved single peaks dominate the low-order region and the background profile stands out in the powder pattern. The critical step of recovering *F*_obs_ is therefore considerably less affected by undesirable model effects. The extraction process may even be further improved, *i.e.* less biased, by upgrading the conventional Rietveld refinement with advanced features such as anharmonic modelling of thermal motion or multipolar modelling of chemical bonding (Fischer *et al.*, 2011 ▶; Kastbjerg *et al.*, 2013 ▶; Bindzus *et al.*, 2014 ▶).

Traditionally, the Pendellösung data on diamond (Takama *et al.*, 1990 ▶) were considered some of the most accurate experimental structure factors ever obtained. However, the ED of diamond attracted renewed attention when Nishibori *et al.* (2007 ▶) published benchmark SPRXD diamond data with a resolution of (sinθ/λ)_max_ = 1.45 Å^−1^ and accuracy comparable with that of the Pendellösung data. The quality of these data enabled Svendsen *et al.* (2010 ▶) to demonstrate that the commonly used *s* ^2^ *p* ^2^ hybridized ED models for the carbon atom are radially defective and that the information about covalent bonding may be preserved in the extraction of *F*_obs_, despite potential bias towards the non-interacting atoms of the IAM. Additionally, this study highlighted the deficiency of the standard Hansen–Coppens (HC) MM [equation (1)] in fitting high-resolution theoretical data. Inspired by this observation, Fischer *et al.* (2011 ▶) formulated the extended Hansen–Coppens (EHC) MM, which abandons the approximation of an inert core. For diamond, the pseudo-atom description assumes the following modified expression
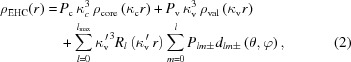
where κ_c_ and *P*_c_ adjust to spherical deformation in the core region. This methodological upgrade results in truly featureless residual maps and eliminates systematic errors in the least-squares determination of the scale and atomic displacement parameters. Moreover, it has the capability to reveal how the innermost ED responds to the formation of chemical interactions. In the case of diamond, covalent bond formation induces a subtle contraction of the core shell (Fischer *et al.*, 2011 ▶). The origin of this is that the 2*s* atomic orbital possesses a radial node and, as a consequence, the deformation of the valence electrons will have an effect on the ED close to the core.

**Figure 6:**
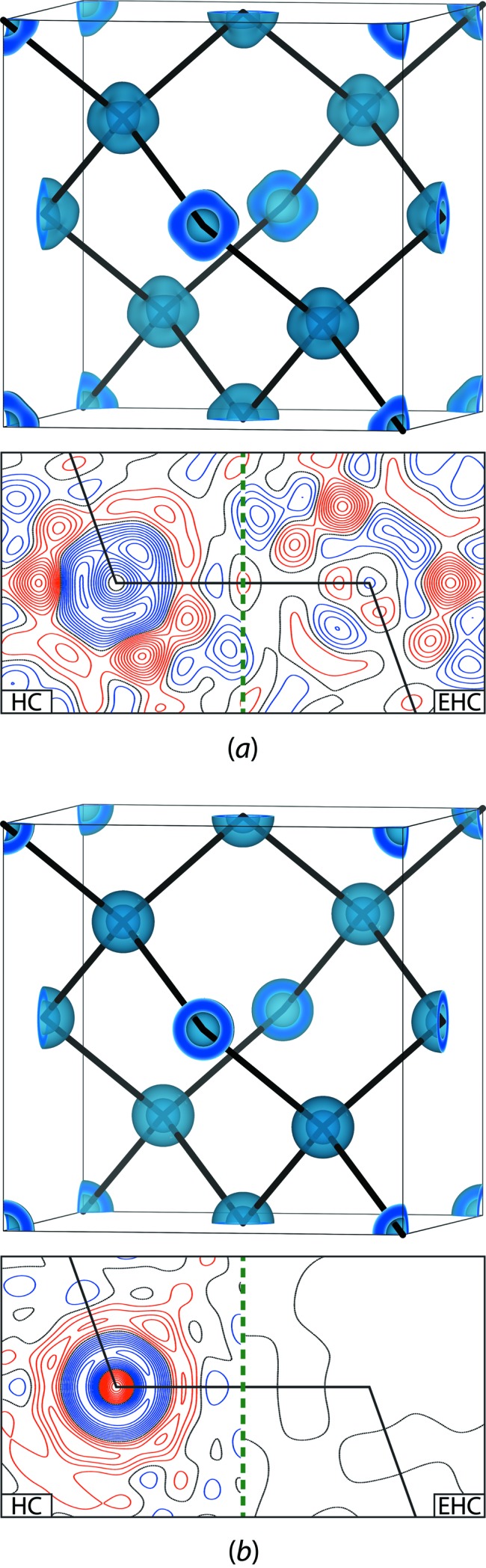
Residual densities for advanced multipolar modelling of diamond based on (*a*) experimental and (*b*) theoretical structure factors to a resolution of (sinθ/λ)_max_ = 1.70 Å^−1^. The isosurface plots and the left side of the contour plots highlight the fact that the assumption of an inert core in the standard HC model results in distinct residual discrepancies near the carbon sites. A correct reconstruction of the innermost CD deformation requires the flexible EHC model (right side). In both models the scale and the atomic displacement parameters are constrained to their true values. Isosurface plots are drawn at −0.06 e Å^−3^ and the contour plots employ a step width of 0.01 e Å^−3^, with positive levels in red, zero in black and negative in blue. Experimental *F*_obs_ are recovered from SPRXD data by multipolar Rietveld refinement. A complete account of this study is found in Bindzus *et al.* (2014 ▶).

Based on newly collected benchmark diamond SPRXD data with an ultra-high resolution of 1.70 Å^−1^, Bindzus *et al.* (2014 ▶) validated the theoretically predicted core shell contraction. The excellent correspondence is illustrated in Fig. 6 ▶. When refining against both the experimental and theoretical structure factors within the standard HC formalism, the core shell contraction materializes as distinct residual density surrounding the carbon sites. The superior fits attained using the EHC model are shown by a much reduced residual density. A key feature of this study was that the mono­atomic diamond structure permitted both deconvolution of the thermal motion and adjustment of the core parameters.

For high-symmetry inorganic systems, SPXRD has evolved into a viable option for performing accurate ED studies. Of particular interest is the opportunity to probe intriguing core polarization phenomena utilizing high-energy SR. The limiting sphere of conventional radiation sources renders them inadequate for this kind of study, but SPXRD data with exceptional resolution can be measured in a matter of minutes at dedicated synchrotron beamlines such as P02.1 (at PETRA III, Germany) and ID22 (at ESRF, France). To facilitate high-order scattering, the former eliminates air scattering by evacuating the flight path all the way from the source to the detector, whereas the latter utilizes a crystal analyser to eliminate inelastic scattering (Hodeau *et al.*, 1998 ▶; Straasø *et al.*, 2013 ▶). Recent technical and methodological developments indicate an exciting future for ED analysis of SPXRD data. This field is moving rapidly beyond benchmark materials such as diamond to explore cases of greater complexity and chemical importance.

## 7. Discussion and outlook   

Many of the examples discussed above include both experimental and theoretical EDs, which naturally raises the question, ‘Are experimental ED determinations becoming obsolete?’ With exponential growth in computing power, the accessible range of complexity for theoretical calculations is ever increasing. This, combined with user-friendly software, allows more researchers to work in this field. However, model ambiguity depending on the basis set and the level of theory applied is unavoidable. Thus, similar to experimental studies, caution should always be exercised when analysing the results. We do not believe that it should be a discussion of one over the other, as both approaches have their own advantages and disadvantages and in general they complement each other well. Small organic molecules are efficiently and readily accessible by high-level theoretical methods. On the other hand, large magnetic MOF structures are currently beyond the reach of accurate high-level calculations. Experimentally, the EDs of these complicated systems are accessible, particularly by using SR. Carefully evaluated experimental EDs will continue to serve as an independent reference for the theoretical ED, and thus a good correspondence between the two methods adds credibility to both.

The XCW method proposed by Jayatilaka (1998 ▶) and Jayatilaka & Grimwood (2001 ▶) is an approach in between fully experimental and fully theoretical work and presents an exciting new way of combining the two. So far, the method is restricted to molecular compounds with only one molecule in the asymmetric unit. If the scope of this method could be expanded to *e.g.* extended structures containing transition metals, it could revolutionize the field by accessing not only the ED but also an experimental wavefunction. This allows a more detailed analysis of the bonding and magnetism in these compounds. The development and benchmarking of this method will depend strongly on the availability of accurate low-temperature diffraction data from SR.

Experimental methods are progressing continually. Today, synchrotron sources are better and brighter than ever, and new sources are being built while existing sources are being upgraded to produce ever higher brilliance. Nonetheless, it is doubtful if these advances will translate directly into ground-breaking new developments in the field of ED determination, as most experiments today are limited by detector capabilities, not by the flux on the sample. Charge-coupled device (CCD) and image-plate (IP) detectors are both mature technologies and only limited evolutionary improvement can be expected in the future. A new emerging technology, hybrid pixel array detectors with direct photon detection, shows great promise for accurate diffraction experiments. These detectors have low noise and a dynamic range much higher than either CCD or IP readers (Broennimann *et al.*, 2006 ▶; Kraft *et al.*, 2009 ▶). Additionally, they have a very fast readout, which facilitates ‘shutterless’ experiments where the crystal is rotated continuously, compared with the sequential oscillation method commonly employed today. This operation method promises lower systematic errors, as timing jitter of the shutter will be completely eliminated and movement of the goniometer motors will be simplified. These effects are especially significant for the very short exposure times commonly employed at synchrotron sources (Diederichs, 2010 ▶). While the macromolecular crystallography community already employs these detectors (Hülsen *et al.*, 2006 ▶; Mueller *et al.*, 2012 ▶), to our knowledge no SR ED determination using these new detectors has been published. However, a low-resolution data set, (sinθ/λ)_max_ ≃ 0.7 Å^−1^, was collected on the antibiotic ceftazidime pentahydrate (Schürmann *et al.*, 2012 ▶). Remarkably, the data set was collected in only 200 s using the shutterless data-collection mode. An invariom model (Hübschle, Luger & Dittrich, 2007 ▶) was refined against the data and, despite the high *R* values of the high-intensity reflections, the experiment illustrates the feasibility and potential of using these detectors for ED research. The data requirements for macromolecular crystallography are indeed very different from the high data quality essential for accurate ED work. As this technology is new, a significant amount of effort needs to be invested in understanding the details of their characteristics and ensuring that these detectors become suitable for ED data collection. A similar effort was invested in the transition from serial detectors to area detectors at the beginning of the 1990s. An example of a correction that is absolutely essential for accurate ED work is the oblique incident angle correction (Zaleski *et al.*, 1998 ▶; Iversen *et al.*, 1999 ▶; Wu *et al.*, 2002 ▶), which went unnoticed by the macromolecular community for a long time due to less demanding data requirements. With regard to hybrid pixel detectors, several problems, in particular those relating to dead-time corrections, high-energy sensitivity, blind inter-pixel areas and integration due to low background and zero-point spread, are expected to present challenges, as well as necessitating adaptations to current software.

Other exciting developments in the field should be mentioned. One such that is particularly noteworthy is the joint refinement of the spin density and the ED, as demonstrated by Deutsch *et al.* (2012 ▶, 2014 ▶). This method utilizes the complementary information in X-ray and neutron diffraction data, in addition to polarized neutron diffraction data, to determine the spin-resolved ED. The joint refinement leads to a more detailed description of the spin density than that from refinement of the polarized neutron data alone. Collecting polarized neutron diffraction data is experimentally challenging and large crystals are required, but in spite of these difficulties, the pioneering work done by Deutsch and co-workers indicates great potential for this method.

Ground-breaking work has been carried out on determining the EDs of crystals in excited states by Pillet *et al.* (2008 ▶), where the ED of an iron spin-crossover compound was determined in its high-spin state. The authors noted some of the problems associated with this type of study, *e.g.* decreased crystal quality introduced by constant cooling and laser irradiation, thus reducing the data quality. While the data quality is inherently lower than regular ED data and this type of experiment may never be routine, it yields important insight into excited-state EDs that are currently beyond reach by theoretical methods for all but isolated molecules (Elliot *et al.*, 2009 ▶). In general, it is expected that more and more ED studies will focus on crystals in perturbed conditions. There has been huge progress in the development of diamond anvil cells for high-pressure crystallography (Boldyreva & Dera, 2010 ▶) and it is anticipated that ED studies at elevated pressure will appear. With improved detector technology at state-of-the-art synchrotron facilities it may also become possible to measure EDs on crystals subjected to external electric or magnetic fields (Fertey *et al.*, 2013 ▶). Clearly, SR measurements will be vital in such developments.

During the last decade, the number of EDs measured with SR has increased greatly and more studies are taking advantage of the compelling properties of SR. As crystallographers we must strive constantly to improve the quality of the data collected. This does not necessarily mean using the newest synchrotron or detector, but rather using the best setup for the question in hand with attention to detail. For well scattering crystals with limited absorption and extinction, the use of a well calibrated stable conventional diffractometer may yield data equivalent to those of a synchrotron experiment. On the other hand, for difficult samples the use of high energy and high intensity is essential to achieve accurate and reliable results. In that sense, the choice of hardware is as essential as the selection of a crystal.

Behind the published results shown here, huge efforts have been invested to optimize synchrotron beamlines for ED work. As mentioned in the introduction, experimental measurement of the ED is in principle quite simple. However, the necessity for extreme data accuracy and precision is very demanding. In that sense, the ultimate performance of any diffraction beamline or instrument is measured by its ability to collect high-quality ED data.
